# Rapid Screening and Preparative Isolation of Antioxidants from *Alpinia officinarum* Hance Using HSCCC Coupled with DPPH-HPLC Assay and Evaluation of Their Antioxidant Activities

**DOI:** 10.1155/2018/3158293

**Published:** 2018-01-31

**Authors:** Lei Fang, Hua Zhang, Jie Zhou, Yanling Geng, Xiao Wang

**Affiliations:** ^1^School of Biological Sciences and Technology, University of Jinan, Jinan 250022, China; ^2^Key Laboratory of TCM Quality Control Technology, Shandong Analysis and Test Center, Jinan 250014, China

## Abstract

An efficient method using high-speed counter-current chromatography (HSCCC) coupled with DPPH-HPLC assay has been developed for rapid screening and preparative isolation of antioxidants from ethyl acetate fraction of *Alpinia officinarum* Hance. Target-guided by DPPH-HPLC assay, two antioxidants, galangin and kaempferide, were targeted and further separated with purities of 99.3% and 98.5% by HSCCC using petroleum ether–ethyl acetate–methanol–water (0.8 : 1 : 1 : 0.8, *v*/*v*) as the solvent system. The antioxidant activities of galangin and kaempferide were further evaluated by measuring their inhibiting effects on superoxide anion radical, hydroxyl radical, and hydrogen peroxide in different luminol chemiluminescence (CL) systems. As a result, galangin and kaempferide both showed potent antioxidant activities. Results of the present study indicated that the combinative method by offline coupling DPPH-HPLC and HSCCC could be widely applied for rapid screening and isolation of antioxidants from complex TCM extract.

## 1. Introduction

*Alpinia officinarum* Hance is a medicinal plant widely distributed in Guangdong and Hainan Province, China. Galangal, the dried rhizomes of *A. officinarum*, is commonly used as both a food additive and a traditional Chinese medicine (TCM) for several centuries in China, because of its health-promoting properties to treat stomach ache, colds, and swelling [1]. Phytochemical investigations indicated that flavonoids, glycosides, and diarylheptanoids are the three groups of important chemical constituents of *A. officinarum* [2]. Further pharmacological research showed that *A. officinarum* possessed a variety of activities such as anti-inflammatory, antioxidant, antibacterial, antiparasitic, and anticancer activities [3–5]. Especially, the flavonoids exhibited significant antioxidant activity and have attracted a great amount of attention [6].

However, the previous isolation of antioxidant flavonoids from complex plant extracts was mainly by means of repeated column chromatography over silica gel, preparative HPLC, which was a time-consuming and labor process and often led to the loss of target antioxidant flavonoids due to dilution effects or decomposition in the procedure of isolation and purification [7–9]. Thus, it is necessary to develop an efficient screening and target-guided separation method to screen and separate the antioxidant flavonoids in this plant. Recently, a useful method using HPLC coupled with DPPH assay has been successfully developed and applied to screen the antioxidants from complex plant extracts [10, 11]. High-speed counter-current chromatography (HSCCC), being as a kind of liquid–liquid partition chromatography, is an optimal choice for the preparative method, which eliminates irreversible adsorption of samples on solid support in conventional column chromatography and offers excellent recovery of target compounds [12, 13]. It has been successfully applied to the separation and purification of different kinds of natural products [14, 15]. Recently, HSCCC coupled with DPPH-HPLC experiment has been successfully applied to screen and isolate antioxidants from certain biological samples [16, 17].

**Figure 1:**
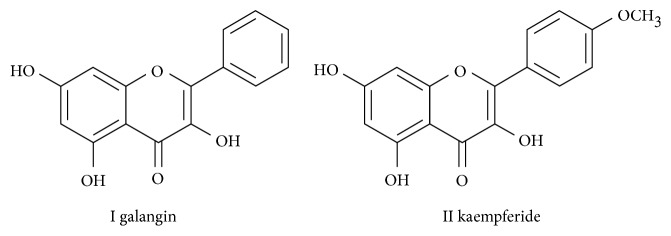
Chemical structures of target-separated antioxidants from *A. officinarum* Hance.

As part of our continuous search for the antioxidants from TCM, ethyl acetate fraction of *A. officinarum* was investigated and showed considerable antioxidant activity under DPPH radical assay. For rapid screening and isolating the antioxidants from the ethyl acetate fraction of *A. officinarum*, an efficient method using HSCCC coupled with DPPH-HPLC assay was developed. Firstly, the ethyl acetate fraction of *A. officinarum* was screened by DPPH-HPLC assay, and two antioxidant flavonoids were targeted. Then, the two flavonoids were target-guided purified using HSCCC and identified as galangin and kaempferide (Figure 1) by electrospray ionization mass spectrometry (ESI-MS) and nuclear magnetic resonance (NMR). Finally, their antioxidant activities were evaluated by different luminol CL systems and DPPH radical assay. This is the first report on rapid screening and preparative isolation of two antioxidants from *A. officinarum* by HSCCC coupled with DPPH-HPLC assay.

## 2. Experimental

### 2.1. Reagents and Materials

Analytical grade solvents used for HSCCC were purchased from Tianjin Chemical Factory (Tianjin, China). Chromatographic grade acetonitrile was used for HPLC and purchased from Siyou Special Reagent Factory (Tianjin, China). All aqueous solutions were prepared with pure water produced by Milli-Q system (Millipore, USA).

The rhizomes of *A. officinarum* Hance were collected in Guangxi, China, and identified by Dr. Jia Li (College of Pharmacy, Shandong University of Traditional Chinese Medicine).

### 2.2. Apparatus

A Model GS10A-2 HSCCC apparatus (Beijing Emilion Science & Technology Co., Beijing, China) with a 230 mL multilayer coil (diameter of 1.6 mm) and with a 20 mL sample loop was employed. The *β* value of the preparative column ranges from 0.5 at the internal to 0.8 at the external (*β* = *r*/*R*, where *r* is the distance from the coil to the holder shaft and *R* is the distance between the holder axis and central axis of the centrifuge). The HSCCC system was also equipped with a Model NS-1007 pump, a Model 8823A-UV Monitor, and a Yokogawa Model 3057 portable recorder.

HPLC was carried out on a Waters Empower system (Milford, MA, USA) including a Model 600 pump, a Model 600 multisolvent delivery system, a Model 996 diode-array detector (DAD), and an Empower workstation. The ESI-MS analyses were performed on an Agilent 1100/MSG1946 (Agilent, CA, USA). The NMR data were recorded on a Varian-600 MHz NMR spectrometer (Varian, Palo Alto, USA).

### 2.3. Preparation of Crude Extracts

**Figure 2:**
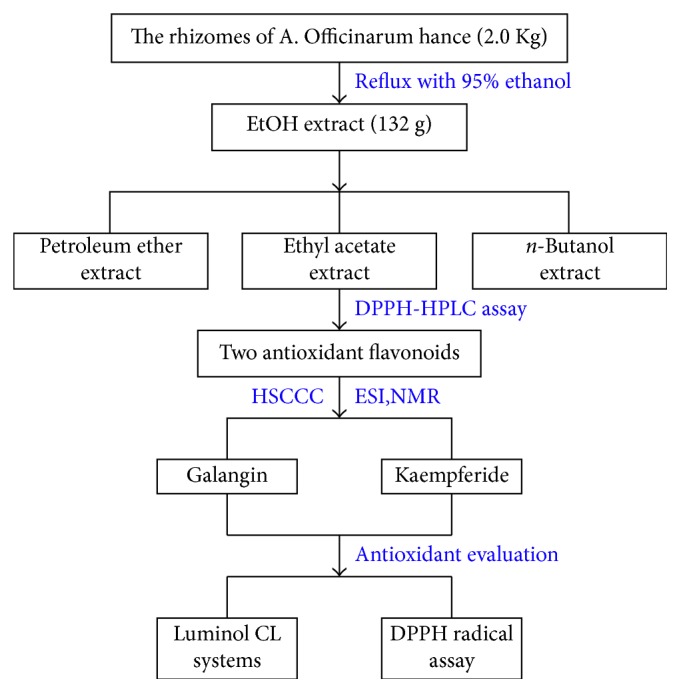
The procedure of screening and preparative isolation of antioxidants from *A. officinarum*.

The rhizomes of *A. officinarum* (2.0 Kg) were pulverized, extracted with 95% ethanol (3 × 15.0 L) under reflux, and evaporated in vacuo to afford a concentrated EtOH extract (132 g). A portion of the crude extract was then suspended in water (500 mL) and successively extracted with petroleum ether (3 × 500 mL), ethyl acetate (3 × 500 mL), and *n*-butanol (3 × 500 mL). This procedure produced ethyl acetate extract (19 g) (Figure 2).

### 2.4. DPPH Radical Assay

The antioxidant activities of the fractions, galangin and kaempferide, were evaluated by DPPH radical assay, which was performed as described in [18]. Analytical results are presented as *x* ± expanded uncertainty in Table 1 [19].

### 2.5. DPPH-HPLC Assay

The DPPH-HPLC assay was performed as described in [17]. In brief, the ethyl acetate fraction of *A. officinarum* (2 mg/ml in methanol, 2 mL) reacted with DPPH (0.26 mg/ml in methanol, 2 mL) for 30 min at room temperature, and then the mixture was subjected to HPLC analysis. The ethyl acetate fraction of *A. officinarum* (4 mg/ml in methanol) was used as a control. The peaks which are reduced or disappeared after reaction will be targeted as potent antioxidants. The HPLC chromatographic separations were accomplished with an Inertsil-ODS-SP column (150 × 4.6 mm, 5 μm) at room temperature. The mobile phase consisted of A (0.2% acetic acid in water) and B (acetonitrile) with the gradient (0–5 min, 55–68% B; 5–15 min, 68% B; 15–30 min, 68–75% B) and the HPLC chromatography was performed at a flow rate of 1.0 ml/min. The effluent was monitored at 254 nm by a photodiode-array detector.

### 2.6. HSCCC Separation

We followed the methods of Lei Fang et al. [20]. The *K* values were determined by HPLC as follows: Approximately 2 mg of crude extract was added to the test tube, and then 2 mL of each phase of the two-phase solvent system was added. After shaken violently for several minutes, an equal volume of each phase was analyzed by HPLC to obtain the partition coefficients. The *K* value was calculated according to the peak area of the compound in the upper phase divided by that in the lower phase.

HSCCC separation was performed as follows: Firstly, the multiplayer coiled column was filled entirely with the stationary phase (upper organic phase). Then, the lower aqueous phase was pumped into the column from the head end at a suitable flow rate of 2 ml/min, while the apparatus was rotated at a speed of 850 rpm. After hydrodynamic equilibrium was reached, the sample solution was injected into the column. A UV detector was applied to continuously monitor the effluent of the column at 254 nm. Each peak fraction was collected according to the elution profile and analyzed by HPLC. The solvents in the column were finally pushed out by pressurized nitrogen gas, and retention of the stationary phase was measured after running.

### 2.7. Analysis and Identification of the Target Compound

The target compound from the preparative HSCCC separation was analyzed by HPLC using the chromatographic separations as described in DPPH-HPLC assay and identified by electrospray ionization mass spectrometry (ESI-MS) on an Agilent 1100/MSG1946 (Agilent, California, USA) and ^1^H and ^13^C NMR spectra on a Varian-600 NMR spectrometer (Varian, Palo Alto, USA).

### 2.8. Antioxidant Capacity Assay by Luminol Chemiluminescence

The antioxidant activity of the target compound was evaluated by measuring the scavenging percentage of superoxide anion, hydrogen peroxide, and hydroxyl radical with luminol chemiluminescence according to the previous reference [21].

#### 2.8.1. Superoxide Anion (O_2_^−^)

0.5 mL sample with different concentrations was added to 4 mL luminol solution, and the mixture was put into detection pool. After injection of 0.2 mL pyrogallol, the change of emission intensity was recorded as a function of time.

#### 2.8.2. Hydrogen Peroxide (H_2_O_2_)

0.5 mL sample with different concentrations was added to 4 mL luminol solution, and the mixture was put into detection pool. After injection of 0.2 mL hydrogen peroxide, the change of emission intensity was recorded as a function of time.

#### 2.8.3. Hydroxyl Radical (HO^·^)

0.5 mL sample with different concentrations and 0.2 mL ferrisulphas were added to 4 mL luminol solution, and the mixture was put into detection pool. After injection of 0.2 mL hydrogen peroxide, the change of emission intensity was recorded as a function of time.

#### 2.8.4. Data Analysis

The scavenging percentage of the target compound was calculated according to the following formula: [(*PA*_Blank_−*PA*_Sample_)/*PA*_Blank_] × 100%. L-ascorbic acid was used as a reference compound, and 5% ethanol was used as the blank. The value of IC_50_ was calculated by Origin 8.0 Version software from the graph plotting inhibition percentage. Analytical results are presented as *x* ± expanded uncertainty in Table 2 [19].

### 2.9. Quality Assurance and Quality Control (QA/QC)

In the DPPH radical assay and antioxidant capacity assay by luminol chemiluminescence, the samples were tested and analyzed together with one analytical blank and one reference compound as the QA/QC sample. Due to the high degree of automation in the above method, three sets were prepared and analyzed at the same time as one batch. The DPPH-HPLC assay served as a procedure of qualitative analysis in the study. For QA/QC purposes, measurement of a target analyte in a set of samples was considered valid only when the RSD of the peak area in the repeatability test must not deviate more than 2.0% (*n* = 3).

## 3. Results and Discussion

### 3.1. Antioxidant Activities of Different Fractions of *A. officinarum*

The DPPH radical assay by measuring the scavenging ability of antioxidants is widely used to evaluate antioxidant activity. The extract of *A. officinarum* was fractionated by solvents with different polarity and afforded the petroleum ether, ethyl acetate, and *n*-BuOH fractions. The different fractions of *A. officinarum* were then evaluated by DPPH radical assay. As shown in Table 1, the ethyl acetate fraction showed the highest capacity to scavenge DPPH radical with the IC_50_ value of 32.9 ± 0.3 μg/mL among all the fractions tested. The results indicated that ethyl acetate fraction of *A. officinarum* was rich in antioxidants.

### 3.2. Screening Antioxidants by DPPH-HPLC Assay

The DPPH-HPLC assay has been used for rapid screening of antioxidants from complex mixtures, especially for TCM extract. In the assay, the peak areas of antioxidants will decrease or disappear in the HPLC chromatogram after spiking with DPPH, while the peak areas for those without antioxidant activities will not change.

**Figure 3:**
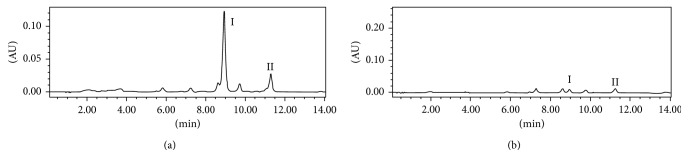
(a) HPLC chromatogram of the ethyl acetate extract from *A. officinarum* Hance; (b) HPLC analysis of the ethyl acetate extract from *A. officinarum* Hance after spiking with DPPH. Experimental conditions: column, Inertsil-ODS-SP column (150 × 4.6 mm I.D., 5 μm); column temperature, 25°C; mobile phase, 0.2% acetic acid-acetonitrile with the gradient (0–5 min, 55–68% B; 5–15 min, 68% B; 15–30 min, 68–75% B); flow rate, 1.0 mL/min; detection, 254 nm; injection volume, 20 μL.

It is the first and crucial step in DPPH-HPLC assay to choose optimal HPLC conditions which allow all compounds in the complex extract to be separated completely. The HPLC conditions were carefully optimized, including different mobile phases with different elution modes, different detection wavelengths, and different flow rates. The results indicated that the optimal mobile phase consisted of A (0.2% acetic acid in water) and B (acetonitrile) with the gradient (0–5 min, 55–68% B; 5–15 min, 68% B; 15–30 min, 68–75% B). The flow rate was 1.0 ml/min, and 254 nm was selected as the detection wavelength. Under optimized HPLC conditions, the ethyl acetate extract of *A. officinarum* was analyzed, and the analytical results are presented in Figure 3.

The ethyl acetate fraction of *A. officinarum* spiking with DPPH was analyzed by HPLC at 254 nm and is shown in Figure 3 in which peaks **I** and **II** almost disappeared after spiking with the DPPH solution, while peak areas of other compounds little changed. The results indicated that only peaks **I** and **II** possessed potent antioxidant activities. Thus, peaks **I** and **II** were targeted and further isolated by HSCCC.

### 3.3. Targeted Separation of Peak **I** by HSCCC

**Figure 4:**
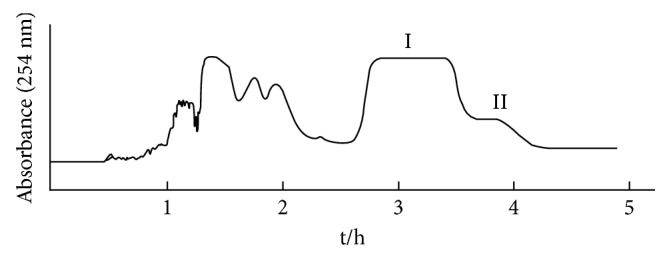
HSCCC chromatogram of ethyl acetate extract of *A. officinarum* Hance. Solvent system: petroleum ether–ethyl acetate–methanol–water (0.8 : 1 : 1 : 0.8, *v*/*v*); revolution speed: 850 r/min; flow rate: 2.0 mL/min; sample size: 270 mg; UV detection wavelength: 254 nm.

**Figure 5:**
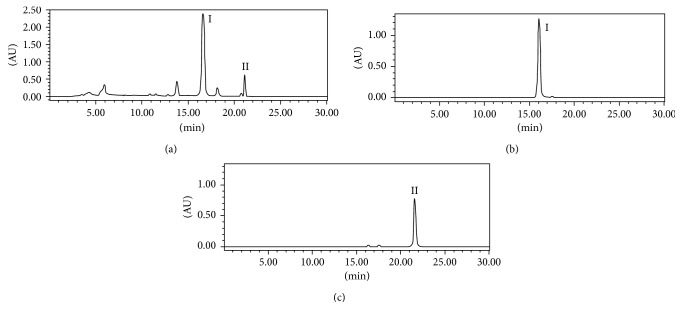
(a) HPLC chromatogram of the ethyl acetate extract from *A. officinarum* Hance; (b, c) HPLC analyses of target-separated antioxidants purified with HSCCC. Experimental conditions: column, Shim-pack VP-ODS column (250 × 4.6 mm I.D., 5 μm); column temperature, 25°C; mobile phase, 0.2% acetic acid-acetonitrile with the gradient (0–5 min, 55–68% B; 5–15 min, 68% B; 15–30 min, 68–75% B); flow rate, 1.0 mL/min; detection, 254 nm; injection volume, 20 μL.

The targeted separation of peaks **I** and **II** was performed by HSCCC. The selection of a suitable two-phase solvent system is the first and critical step for a successful separation using HSCCC. The suitable solvent system should provide an ideal range of partition coefficient (*K*, 0.5–2) for the target antioxidant [13]. To our best knowledge, more than 60% of flavonoid derivatives were isolated by HSCCC with the solvent system of petroleum ether–ethyl acetate–methanol–water [22]. Then, several kinds of solvent systems based on petroleum ether–ethyl acetate–methanol–water were assessed, and the values of *K* for **I** and **II** were conducted by varying volume ratios. It can be found that the *K* values of 0.88 and 1.45 in the solvent systems with the volume ratio of 0.8 : 1 : 1 : 0.8 (*v/v*) were suitable for separation of the target compounds. As shown in Figure 4, good resolution and acceptable separation time could be obtained when petroleum ether–ethyl acetate–methanol–water (0.8 : 1 : 1 : 0.8, *v/v*) was used as the two-phase solvent system. The fractions of HSCCC were collected and analyzed by HPLC (Figure 5). As a result, 107 mg of compound **I** and 29 mg of compound **II** with the purities of 99.3% and 98.5%, respectively, were separated and purified in one step by the preparative HSCCC from 270 mg of the ethyl acetate extract. The retention of the stationary phase was 74.0%, and the separation time was within 5 h in each separation run. The HPLC chromatograms of crude extract and the pure compounds are shown in Figure 4. This is the first report on screening and targeted isolation of the major antioxidant from *A. officinarum* by DPPH-HPLC combined with HSCCC, which would give an excellent example to separate active compounds from complex mixtures.

### 3.4. Structual Identification of the Target-Separated Antioxidants

The structural identification of the separated antioxidants was carried out by ESI-MS, ^1^H NMR, and ^13^C NMR spectra as follows:

Compound **I**: ESI-MS *m/z*: 271 [M + H]^+^. ^1^H NMR (DMSO-*d*_5_, 600 MHz) *δ*: 8.02 (2H, *d*, *J* = 7.8 Hz, H-2′, 6′), 7.54 (3H, *m*, H-3′, 4′, 5′), 6.52 (1H, *s*, H-8), 6.44 (1H, *s*, H-6). ^13^C NMR (DMSO-*d*_5_, 150 MHz) *δ*: 93.8 (C-8), 98.9 (C-6), 103.7 (C-10), 128.0 (C-3′, 5′), 128.9 (C-2′, 6′), 130.4 (C-4′), 131.5 (C-1′), 137.5 (C-3), 146.2 (C-2), 156.8 (C-9), 161.2 (C-5), 164.6 (C-7), 176.8 (C-4). According to the literature, compound **I** was identified as galangin [23].

Compound **II**: ESI-MS *m/z*: 301 [M + H]^+^. ^1^H NMR (DMSO-*d*_5_, 600 MHz) *δ*: 8.12 (2H, *d*, *J* = 7.8 Hz, H-2′, 6′), 7.18 (2H, *J* = 7.8 Hz, H-3′, 5′), 6.42 (1H, *s*, H-8), 6.24 (1H, *s*, H-6), 3.85 (3H, *s*, OCH_3_). ^13^C NMR (DMSO-*d*_5_, 150 MHz) *δ*: 94.0 (C-8), 99.1 (C-6), 103.8 (C-10), 114.7 (C-3′, 5′), 129.8 (C-2′, 6′), 160.1 (C-4′), 124.1 (C-1′), 136.7 (C-3), 146.9 (C-2), 156.8 (C-5), 161.3 (C-9), 164.8 (C-7), 176.6 (C-4). According to the literature, compound **II** was identified as kaempferide [23].

### 3.5. Antioxidant Activities of Target-Isolated Compounds

The antioxidant activities of target-isolated compounds, galangin and kaempferide, from the ethyl acetate fraction of *A. officinarum* were measured by luminol chemiluminescence and DPPH radical assay in comparison with rutin as positive antioxidant. As shown in Table 2, galangin and kaempferide showed inhibitory effects on different luminol CL systems including superoxide anion radical, hydroxyl radical, and hydrogen peroxide. Meanwhile, galangin and kaempferide exhibited effective antioxidant activities against DPPH with IC_50_ values of 4.2 ± 0.03 μM and 7.8 ± 0.04 μM, respectively, which were in accordance with the DPPH-HPLC experiment.

## 4. Conclusions

In this paper, an efficient method using HSCCC combinative with DPPH-HPLC assay was successfully developed for rapid screening and preparative isolation of antioxidants from ethyl acetate fraction of *A. officinarum* Hance. Using this method, two antioxidants, galangin and kaempferide, were rapidly targeted and further separated with purities of 99.3% and 98.5%, respectively, which also showed significant antioxidant activities by luminol chemiluminescence and DPPH radical assay. The innovative potential of the method is that the antioxidants in the complex extract can be rapidly screened from the complex extract by DPPH-HPLC assay and then target-guided purified by HSCCC. The results of this study indicate that HSCCC coupled with DPPH-HPLC assay has a broad applicability and is a very powerful and effective method for rapid screening and separation of the antioxidants from TCM.

## Figures and Tables

**Table 1:** Antioxidant activities of fractionations of different polarities and target-separated antioxidants in DPPH radical assay.

Samples	DPPH (IC_50_, μM)
Petroleum ether fraction	>100
Ethyl acetate fraction	32.9 ± 0.3
*n*-BuOH fraction	74.5 ± 0.6
Galangin	4.2 ± 0.03
Kaempferide	7.8 ± 0.04
Rutin^a^	3.9 ± 0.03

^a^Used as control.

**Table 2:** Antioxidant activities of ethyl acetate fraction and target-separated antioxidants on different luminol CL systems.

Samples	Chemiluminescence systems (IC_50_, 10^−3^ mg/mL)		
	O_2_^−^	H_2_O_2_	HO^·^
Ethyl acetate fraction	5.89 ± 0.56	1.09 ± 0.08	1.31 ± 0.07
Galangin	1.26 ± 0.14	4.93 ± 0.32	3.54 ± 0.22
Kaempferide	2.35 ± 0.25	6.72 ± 0.47	4.68 ± 0.29
Rutin^a^	3.11 ± 0.34	1.92 ± 0.11	1.37 ± 0.06

^a^Used as control.
